# Signal Transducers and Activators of Transcription (STAT) Regulatory Networks in Marine Organisms: From Physiological Observations towards Marine Drug Discovery

**DOI:** 10.3390/md13084967

**Published:** 2015-08-07

**Authors:** Jin-Young Lee, Barbora Orlikova, Marc Diederich

**Affiliations:** 1 Department of Pharmacy, College of Pharmacy, Seoul National University, Seoul 151-742, Korea; E-Mails: snujylee@snu.ac.kr (J.-Y.L.); barbora.orlikova@lbmcc.lu (B.O.); 2 Laboratoire de Biologie Moléculaire et Cellulaire du Cancer, Hôpital Kirchberg 9, rue Edward Steichen, Luxembourg 2540, Luxembourg

**Keywords:** anti-cancer, drug discovery, JAK/STAT signaling

## Abstract

Part of our ocean’s richness comes from its extensive history of supporting life, resulting in a highly diverse ecological system. To date, over 250,000 species of marine organisms have been identified, but it is speculated that the actual number of marine species exceeds one million, including several hundreds of millions of species of marine microorganisms. Past studies suggest that approximately 70% of all deep-sea microorganisms, gorgonians, and sea sponges produce secondary metabolites with anti-cancer activities. Recently, novel FDA-approved drugs derived from marine sponges have been shown to reduce metastatic breast cancer, malignant lymphoma, and Hodgkin’s disease. Despite the fact that many marine natural products have been shown to possess a good inhibition potential against most of the cancer-related cell signaling pathways, only a few marine natural products have been shown to target JAK/STAT signaling. In the present paper, we describe the JAK/STAT signaling pathways found in marine organisms, before elaborating on the recent advances in the field of STAT inhibition by marine natural products and the potential application in anti-cancer drug discovery.

## 1. Introduction

Cancer mortality rates remain high despite tremendous research efforts and innovative clinical trials with new drug candidates. Many of these compounds were from natural origins and, according to the World Health Organization (WHO), over 80% of the global population depends on plant-derived herbal medicines for their healthcare, especially in developing countries [1]. Worldwide, over 50% of the anti-cancer pharmaceutical market is based on derivatives from natural resources and their synthetic metabolites, including derivatives from plants, insects, and marine life [2,3]. Among them, almost 60% of naturally-derived anti-inflammatory [4,5] and anti-cancer drugs are approved for therapeutic applications [6]. Fruits, vegetables, and herbs are very basic resources which have been used for their anti-oxidant and anti-aging properties by healthcare researchers for a long time [7], based on their contents of vitamins, fiber, antioxidants, phenolic and carotenoid compounds. Development of therapeutic drugs, such as penicillin, aspirin, and cyclosporine, which are derived from natural plant chemicals and soil organisms, dates back many decades. Thus, research and development of naturally-derived products is still highly interesting and promising [8].

Marine-derived natural products have also been found to be highly interesting for the anti-cancer drug industry even though they are not yet sufficiently exploited [9,10,11,12]. Although oceans cover approximately 70% of our planet, there are still huge untapped marine resources available for the discovery of future therapeutic applications. Approximately 95% of all oceans reach depths of over a thousand meters, and these depths hold promises for discovery and development of bioactive compounds [2,3,13,14,15]. Previous studies have shown that the ocean has yet unexplored regions of biological diversity with various organisms residing in extreme environments without oxygen, light and under high pressure [16]. To live under these conditions, organisms must have both physiological and biological adaptations for survival [10]. These adaptations often involve modification of metabolic pathways and methods of genetic regulation, and these condition-acclimatized products should prove to be very valuable for human health.

**Figure 1:**
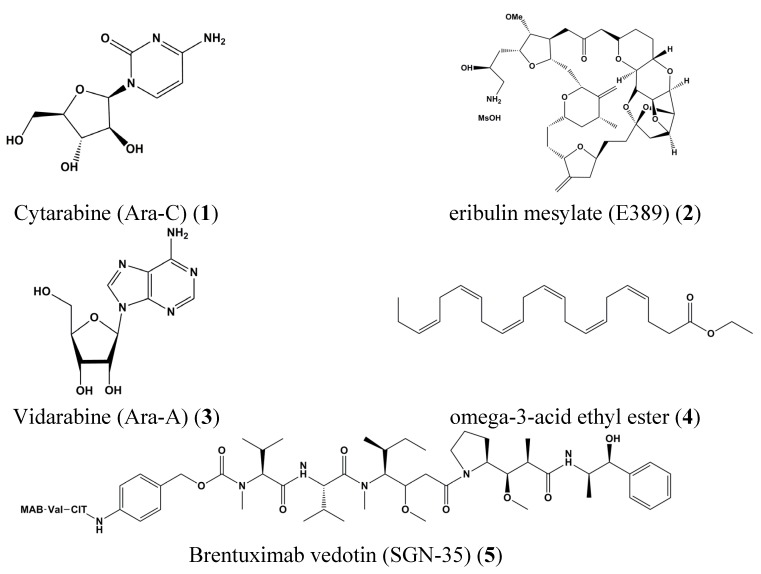
Chemical structures of Cytarabine (Ara-C) (**1**), Eribulin mesylate (E389) (**2**), Vidarabine (Ara-A) (**3**), omega-3-acid ethyl ester (**4**) and Brentuximab vedotin (SGN-35) (**5**).

Furthermore, important FDA-approved drugs were already derived from sponges, for example, the leukemia drugs Cytarabine (Ara-C) (**1**) and eribulin mesylate (E389) (**2**), which reduce metastatic breast cancer as well as the herpes simplex virus drug, Vidarabine (Ara-A) (**3**). Other derivatives from fish like omega-3-acid ethyl ester (**4**) are active on hypertriglyceridemia. Brentuximab vedotin (SGN-35) (**5**) originally from mollusk associated cyanobacteria affects anaplastic large T-cell systemic malignant lymphoma, and Hodgkin’s disease [17] (Figure 1).

Whereas marine drugs were shown to affect basically all hallmarks of cancer [13] and even to contribute to epigenetic reprogramming [15], selected cell signaling pathways remain to be further investigated. While many compounds were shown to affect nuclear factor-κB (NF-κB) signaling [18], less is known about the impact of natural compounds of STAT-signaling in cancer [19]. Moreover, it appears that STAT signaling in marine organisms is also an emerging field of research so that we suggest here to review both the impact of endogenous STAT signaling as an essential function in marine organisms followed by the description of anticancer applications of selected anti-STAT marine compounds. This topic will be highlighted by recent advancements in marine compound drug discovery.

## 2. Past and Present of Drug Discovery in the Field of Cancer and Marine Influences

According to classical pharmacology statements by *Pedanius Dioscorides* in 78 A.D. in *De Materia Medica*, ancient Western societies used thousands of natural substances from medicinal herbs, and these have been found to be very useful in industrial and therapeutic drug applications [7]. Nature-derived materials have been the starting point for the development of most chemotherapeutic drugs over the last 40 years [11].

These natural compounds come with outstanding structural diversity and act most likely as physiological regulators with often-unexplored functions. Nevertheless, they serve as basic templates for the development of drugs with biological properties, through interdisciplinary studies using ecology, biology, pharmacology, and chemistry. These interdisciplinary studies aim to create novel bioactive compounds that can then by synthesized, purified, and characterized for therapeutic bioactivities. In 1960, the National Cancer Institute (NCI) began a large-scale project aiming to discover novel anti-cancer compounds [20,21]. As a result, 35,000 herb samples were screened in cancer cell lines, and *Taxus brevifolia*-derived Taxol (**6**) was developed as part of this project between 1960 and 1982 [22]. Taxol (**6**) is an FDA-approved anti-cancer agent to treat ovarian cancer, breast cancer, lung cancer, and gastric adenocarcinoma [23,24,25].

**Figure 2:**
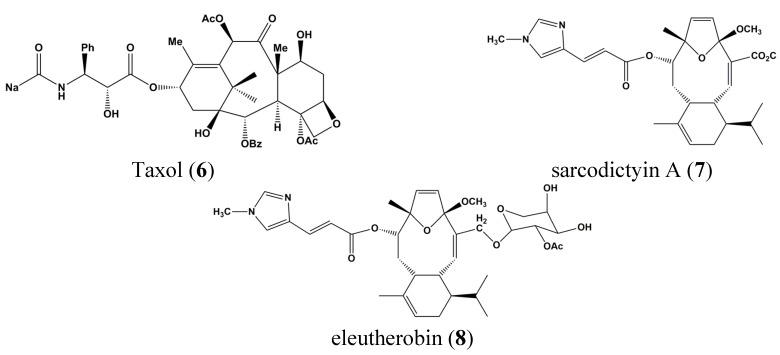
Chemical structures of Taxol (**6**), sarcodictyin A (**7**) and eleutherobin (**8**).

In the 1950s, several compounds derived from *Cryptotethya crypta* from Caribbean sponges showed initially anti-viral effects. Based on these bioactivity assays, Ara-C (cytosine arabinoside) (**1**) was developed several years later and it became an FDA-approved drug [26]. Since then, marine-derived natural chemical compound research has come a long way. Sarcodictyins (**7**) and eleutherobin (**8**) were first isolated from marine corals in 1987 and 1994 and were found to possess anti-cancer activities, with efficacies 50 times greater than Taxol (**6**) (Figure 2).

In 1985, the NCI started a new project in which compounds from marine-derived microorganisms, plants and animals were tested on a panel of 60 human cell lines, including lung, skin, ovary, breast, brain, prostate, kidney, and colon cancer as well as leukemia [27]. By now, at least seven marine-derived compounds are FDA-approved pharmaceutical drugs, and two compounds are undergoing phase III clinical trials. Five other compounds are in phase II clinical trials, three compounds are in phase I/II, and 13 compounds are in phase I. Targets of these derivatives include a wide range of diseases, including cancer and compounds, which will be described in the next section.

## 3. Clinical Trials of Marine-Derived Anticancer Drugs

**Figure 3:**
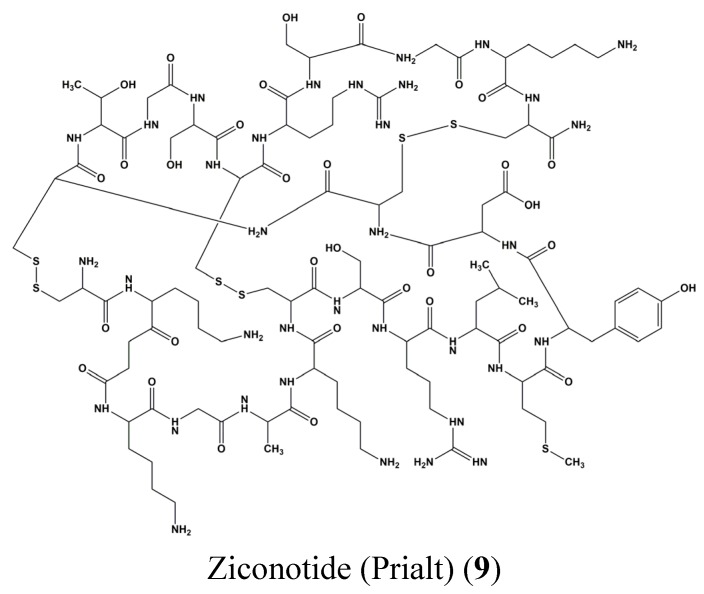
Chemical structure of Ziconotide (Prialt) (**9**).

Cytarabine (arabinosyl cytosine or cytosine arabinoside, Ara-C) (**1**) (Figure 1), vidarabine (arabinofuranosyladenine or adenine arabinoside, Ara-A) (**3**) (Figure 1), and ziconotide (Prialt) (**9**) (Figure 3) are marine-derived drugs approved by the Food and Drug Administration (FDA) [28,29].

Cytarabine **(1)** is a synthetic compound from *Tethya crypta*, a Caribbean sponge [11]. Cytarabine (**1**) arrests the cell cycle by working as an S-phase antimetabolite-like cytotoxic drug and it causes inhibition of Deoxyribonucleic Acid (DNA) polymerase and thus synthesis. Cytarabine (**1**) (Figure 1) was developed from both Cytosar-U and Depocyt, which received FDA approval in 1969. Cytarabine (**1**) is used for treatment of meningeal leukemia, myelogenous leukemia, acute myelocytic leukemia (AML), and acute lymphocytic leukemia (ALL) [30,31].

**Figure 4:**
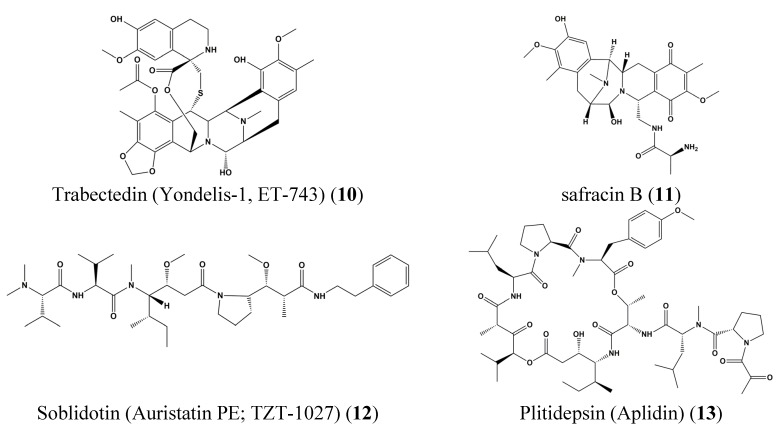
Chemical structures of Trabectedin (Yondelis-1, ET-743) (**10**), Safracin B (**11**), Soblidotin (Auristatin PE; TZT-1027) (**12**) and Plitidepsin (Aplidin) (**13**).

European Medicines Agency (EMEA) also recently approved Trabectedin (Yondelis-1, ET-743) (**10**), discovered in *Ecteinascidia turbinata* in the Mediterranean and Caribbean. Originally, this synthetic compound was developed from safracin B (**11**) with over 90 steps of synthesis and multiple semisynthetic processes including spiro tetrahydroisoquinoline formation, esterification, Curtius rearrangement, carbinolamine formation, and Mannich bisannulation [32]. Trabectedin (**10**) is capable of arresting cell cycle progression in G2/M phase, and it induces p53-independent apoptosis [33]. This molecule, with the trade name Yondelis, was the first compound approved by the European Union as a marine-derived anticancer agent [34] and is effective in treating relapsed platinum-sensitive ovarian cancer [35]. Soblidotin (Auristatin PE; TZT-1027) (**12**), Plitidepsin (Aplidin) (**13**) from tunicate and Tetrodotoxin (tectin) from puffer fish are in Phase III clinical trials in the USA, and plitidepsin (Aplidin) (**13**) is also in active clinical trials in the EU. Soblidotin (Auristatin PE; TZT-1027) (**12**) is a synthetic compound derived from dolastatin and is a vascular disrupting agent, which targets vascular tumors through the inhibition of tubulin activation [36,37]. Clinical trials for this molecule were conducted in USA, Japan, and EU, but after Phase I and II clinical trials, the licensing permissions were terminated (Figure 4).

**Figure 5:**
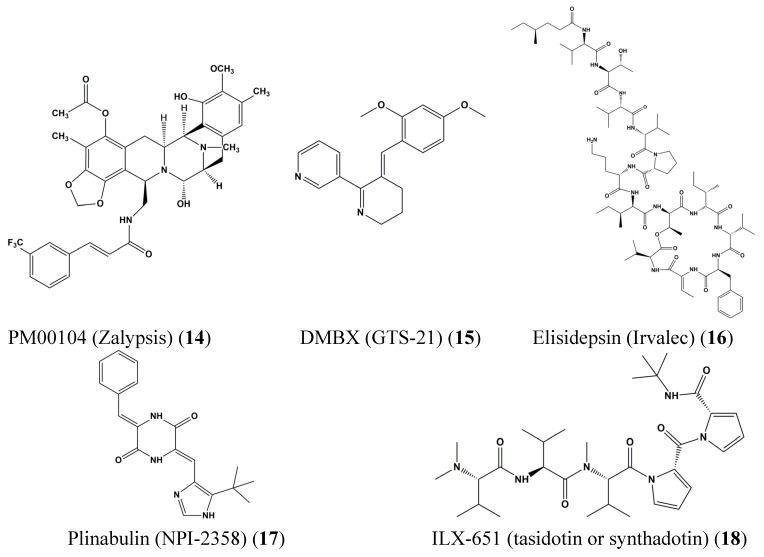
Chemical structures of PM00104 (Zalypsis) (**14**), DMXBA (GTS-21) (**15**), elisidepsin (Irvalec, PM02734) (**16**), plinabulin (NPI-2358) (**17**) and ILX-651 (tasidotin or synthadotin) (**18**).

Marine-derived natural compounds in phase II clinical trials include PM00104 (Zalypsis) (**14**), DMXBA (GTS-21) (**15**), Elisidepsin (Irvalec, PM02734) (**16**), Plitidepsin (Aplidin) (**13**), Plinabulin (NPI-2358) (**17**), and ILX-651 (tasidotin or synthadotin) (**18**) (Figure 5).

DMXBA (GTS-21) (**15**) is derived from anabaserine extracted from nemertines, a phylum of carnivorous, mainly marine worms. DMXBA (GTS-21) (**15**) shows activation of anti-inflammation mechanisms that are modulated through effects on macrophage specific receptors [38]. Recently, studies demonstrated improvement of cognition fBRC [39,40] so that the drug is developed as a novel anti-Alzheimer’s disease compound. PM00104 (Zalypsis) (**14**) is isolated from mucus of renieramycins and *Jorunna funebris*-derived from tunicates and marine sponges [41]. PM00104 (Zalypsis) (**14**) induces S-phase cell cycle arrest and apoptosis through induction of DNA double strand breakage in various cancer cells [42] with powerful antitumor functions in prostate, breast, and renal cancer as well as in hematological diseases. This compound was developed as Zalypsis and is undergoing phase II clinical trials.

**Figure 6:**
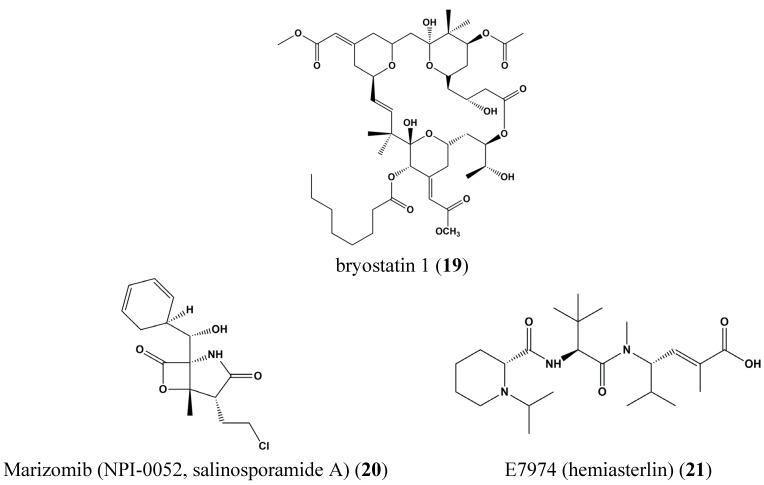
Chemical structures of bryostatin 1 (**19**), Marizomib (NPI-0052, salinosporamide A) (**20**) and E7974 (hemiasterlin) (**21**).

Several marine natural compounds are in phase I clinical trials, including bryostatin 1 (**19**), Marizomib (NPI-0052, salinosporamide A) (**20**), and E7974 (hemiasterlin) (**21**), identified in marine sponges [43]. These induce tumor cell apoptosis, through an antimitotic tubulin-based mechanism [43]. Although the tubulin-mediated agent binds β-tubulin, hemiasterlin predominantly binds to α-tubulin, and has been found to abrogate carcinogenesis in esophageal and prostate cancer [43]. Marizomib (NPI-0052, salinosporamide A) (**20**) is a marine-derived compound from marine *Salinispora tropica* and is a selective proteasome inhibitor [44,45,46]. By inhibiting the proteasome, non-lysosomal proteins are degraded, which could be a target for the treatment of cancer. Marizomib (NPI-0052, salinosporamide A) (**20**) is also in phase I clinical trials for treatment of lymphomas, leukemia, multiple myeloma, and various solid tumors (Figure 6).

## 4. STAT Signaling in Health and Disease

### 4.1. JAK/STAT Signaling Pathways

Single transducer and activator of transcription (STATs) are critical mediators of functional responses and specificity in cytokine signaling. After activation of a receptor complex, STATs are phosphorylated on a conserved tyrosine residue, which induces dimerization, nuclear translocation, and DNA binding and leads to the induction of cytokine-responsive genes [47,48]. The STAT pathway is activated by phosphorylation of cytokines such as interferon (IFN) and interleukin (IL) binding to their specific receptors on the cell surface. In addition to ILs or IFNs, diverse cytokines are now known to trigger STAT activation. These cytokines bind various cytokine receptors that are associated with the Janus kinase (JAK) family [49,50]. There are four types of mammalian JAKs, tyrosine kinases 2 (TYK2), JAK1, JAK2 and JAK3. After ligand binding, receptor and JAK complexes are phosphorylated, which leads to the assembly and phosphorylation of STATs.

Transcriptional activity and their functional roles are required for dimerization of STATs through phosphorylation of particular tyrosine residues, which promote and mediate binding of regulatory receptors with the formed phosphorylated complex [51]. The c-terminal domain of STAT proteins contains the tyrosine-specific phosphorylation site while the n-terminus controls the DNA binding activity. Phosphorylation is regulated by STAT dimerization via SH2 domain interaction [47,48,52].

**Figure 7:**
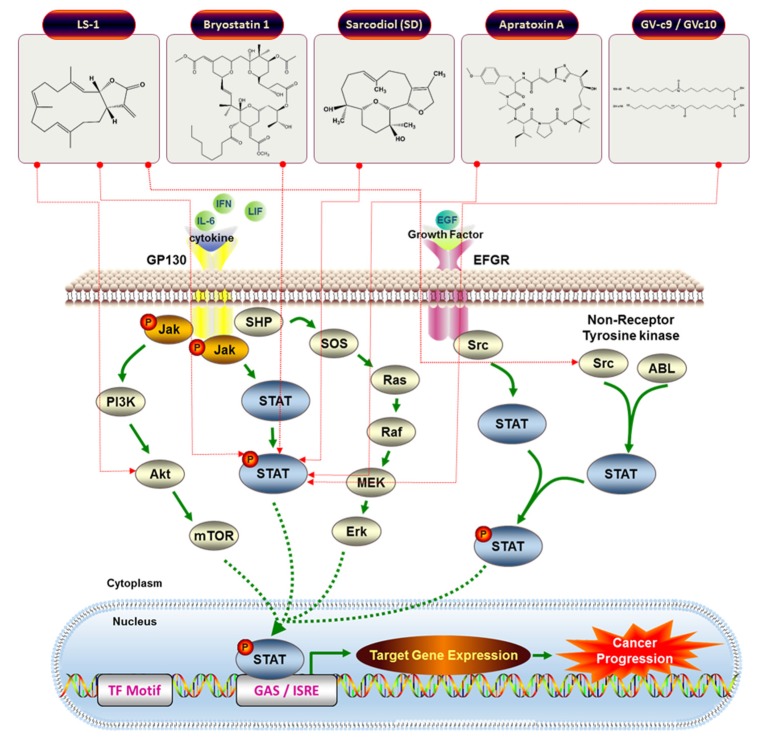
Schematic models for JAK/STAT cell signaling pathway and the inhibitory actions for JAK/STAT target anticancer drugs.

STAT proteins were initially discovered as interferon (IFN) regulated genes in the 1990s [47,48,52]. STATs are composed by seven structurally distinguished members in mammals: STAT1, STAT2, STAT3, STAT4, STAT5a, STAT5b, and STAT6 [53]. These molecules comprise cytoplasmic transcription factors such as cytokine, hormone, and growth factor signal transduction. Additionally, STATs have functions on their downstream effectors [53,54,55,56,57,58,59]. STAT proteins modulate various biological cellular processes including fetal development, organogenesis, apoptosis, growth, differentiation, immune system, and inflammation [54,58,60,61,62,63,64,65,66,67]. STAT proteins often exist as monomers in the cellular cytoplasm, but form dimers through SH2 interactions after tyrosine phosphorylation by ligand stimulation [51]. These complex molecules then translocate into the nucleus to promote transcriptional functions. In tumorigenesis, activated STATs are linked to constitutive activation of tyrosine kinases, including Jak, Break point cluster–Abelson (Bcr-Abl), Epidermal growth factor receptor (EGFR), Src. Selected natural compounds were shown to interfere with JAK/STAT signaling [19,68,69,70] (see Figure 7 for a comprehensive overview).

### 4.2. JAK/STAT Signaling in Marine Organisms

The Jak/Stat pathway is considered essential for immune and anti-inflammatory defense. Accordingly, it is not astonishing to see this signaling pathway appearing together with the adaptive immune system [71] during early vertebrate development. According to Vogl and coworkers, Stats exist in metazoans, choanoflagellates and slime molds. The same authors describe Jaks in bilaterians but absent in mollusks, round- and flatworms. They conclude that “the Jak-Stat pathway evolved at the base of the bilaterians, but has been lost in some invertebrate groups” [72].

So far, a number of studies have reported on the physiological function and existence of an interferon/antiviral response in marine organisms. For instance, IFNs, with structural and functional properties similar to mammalian type I IFNs, were cloned from various types of fish, including Atlantic salmon, channel catfish, pufferfish, zebrafish and other teleost fish [73]. An important question was about the physiological role of these genes and whether they were required for anti-viral defense of marine organisms related to JAK/STAT activation. Interestingly, Santos *et al.* discovered a type-1 cytokine receptor (Japanese flounder glycoprotein 130 homologue; JfGPH) with JAK and STAT 3 binding motifs in the cytoplasmic region. These early results suggested a mediatory role for JAK/STAT signal pathway in fish, so that the authors hypothesized a role in immune response, and reproduction/development [74]. Later suppressors of cytokine signaling 2 (SOCS2) from *Eriocheir sinensis* (EsSOCS2) were shown to be inducible by bacteria so that Zhang *et al.* concluded that this pathway was most likely involved in the immune defense responses in *E. sinensis* [75]. In addition, the economically important large yellow croaker (*Pseudosciaena crocea*) suffers by outbreaks of marine bacteria including *Aeromonas hydrophila*. Here, Mu *et al.* showed that inflammatory response might play an important role in the early stages of fish infection. The authors validated that signaling cascades such as the Toll-like receptor, JAK/STAT, and MAPK pathways to be regulated by *A. hydrophila* infection and to play essential roles in large yellow croaker immune response to bacterial infection [76]. Similarly, turbot *Scophthalmus maximus* SOCS homologue (SmSOCS3) is a cytokine-inducible suppressor of pro-inflammatory cytokine signaling in HK macrophages. Regulated expression of SmSOCS3 is essential for innate immune response against bacterial infection [77].

Li *et al.* published that the transcription of STAT in shrimp was regulated by White spot syndrome virus (WSSV) infection allowing the authors to hypothesize the existence of such a regulatory pathway in shrimp, which would be responsive to viral infection [78]. Many other marine invertebrates with economic importance were characterized concerning their JAK/STAT signaling components: SOCS-2 from pearl oyster *Pinctada fucata* plays a regulatory role against the stimulation [79]; a member of the STAT4 family was discovered in rock bream (*Oplegnathus fasciatus*) (RbSTAT4) and its regulation under pathological stimuli was investigated [80]; Janus kinase (designated as LvJAK) gene was cloned and characterized from whiteleg shrimp *Litopenaeus vannamei* [81]. These examples demonstrate the essential function of JAK/STAT like functions in marine invertebrates so that discovery of compounds interfering with these mechanisms also from marine organisms should be expected, considering the biochemical warfare described so far between marine organisms related to space and nutrients as we previously reported [9].

### 4.3. JAK/STAT-Inhibiting Anticancer Drugs from Marine Organisms

A large number of natural anti-cancer drugs target control of cell signaling pathways involved in carcinogenesis, which is triggered by improper multiple cellular processes including PI3k/Akt, mTOR, NF-κB or JAK/STAT signaling (see Table 1 for details).

**Table 1:** Overview of the effects of marine natural compounds on STAT family proteins.

*N*	Compound(s)	Disease model	Cell lines	Effect observed	STAT protein	Reference
1	Cytarabine (Ara-C)	Leukemia	K562	Inhibition of STAT5 tyrosine phosphorylation	STAT5	[26]
15	DMXBA (GTS-21)	Preadipocyte	3T3-L1	Reduction ASP-mediated chemokine MCP-1 secretion	STAT3	[38]
19	Bryostatin 1	Blood cancer	Chronic lymphocytic leukemia isolates cells	Induction of tyrosine phosphorylation, DNA binding of STAT1	STAT1	[70]
22	(1*S*,2*S*,3*E*,7*E*,11*E*)-3,7,11,15-Cembratetraen-17,2-olide (LS-1)	Colon cancer	HT-29	Disruption of mitochondrial membrane potential, ROS generation, Cell cycle arrest, De-phosphorylation of STAT3	STAT3	[71]
23	Sarcodiol (SD)	Skin cancer	B_16_F_10_	Inhibition of de novo DNA Synthesis, Induction of DNA fragmentation, Inhibition of STAT3	STAT3	[72]
24	Apratoxin A	Osteosarcoma, Breast cancer	U2OS, MCF7	Inhibition of STAT3 tyrosine phosphorylation. Gp130 degradation	STAT3	[73]
25	GV-c9, and GV-c10	Macrophage	Raw 264.7	Inhibition of inflammatory markers (IL-6, TNF-alpha, and nitric oxide)	STAT1	[74]

Even though not many compounds of marine origins were shown to possess activity against JAK/STAT signaling, several drugs targeting the regulation of these pathways are undergoing clinical trials for FDA approval. Bryostatin 1 (**19**) (Figure 6) is isolated from *Bugula neritina*, a bryozoan. These molecules were tested in over 80 clinical trials for disease treatment and were shown to bind to protein kinase C without carcinogenesis promoting activity. According to Battle *et al.*, bryostatin-treated chronic lymphocytic leukemia (CLL) cells show induction of protein kinase C and in turn activation of STAT1, which is essential for CLL differentiation. This research concludes that bryostatin 1 (**19**) promotes MAPK activation, which then induces the production of IFN-γ triggering JAK/STAT1 signaling [82].

**Figure 8:**
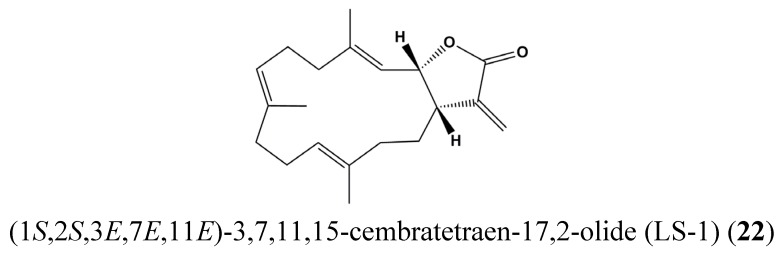
Chemical structure of (1*S*,2*S*,3*E*,7*E*,11*E*)-3,7,11,15-cembratetraen-17,2-olide (LS-1) (**22**).

Hong *et al.* [83] observed that the marine cembrenolide diterpene (1*S*,2*S*,3*E*,7*E*,11*E*)-3,7,11, 15-Cembratetraen-17,2-olide (LS-1) (**22**), possessed antiproliferative and cytotoxic potential in colon cancer cells *via* a reactive oxygen species (ROS)-dependent mechanism. Treatment of HT-29 cells with LS-1 **(22**) (Figure 8) resulted in apoptosis induction *via* the intrinsic mitochondrial pathway. This compound also induced phosphorylation of c-Jun N-terminal kinase (JNK) and dephosphorylation of STAT-3. These findings underline the anticancer efficacy of LS-1 indirectly involving inhibition of STAT3 [83].

**Figure 9:**
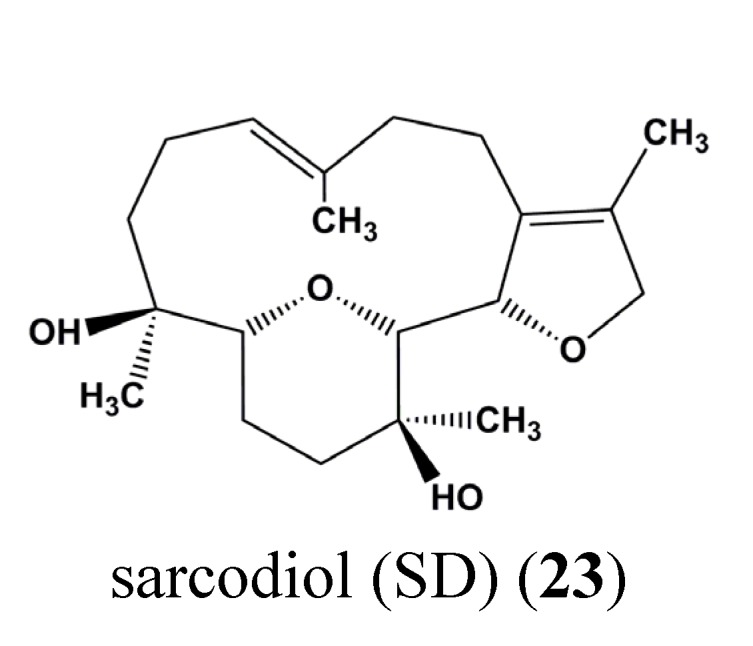
Chemical structure of sarcodiol (SD) (**23**).

Sarcodiol (SD) (**23**) (Figure 9) is a semi-synthetic derivative of sarcophine, a marine natural product. The authors show that SD (**23**) inhibits the *de novo* DNA synthesis and enhances fragmentation of DNA. Interestingly, SD (**23**) inhibits the expression levels of STAT-3 and cyclin D1. SD (**23**) treatment also enhances cellular levels of tumor suppressor protein 53 (p53) and induces caspase-dependent cell death mechanisms [84].

**Figure 10:**
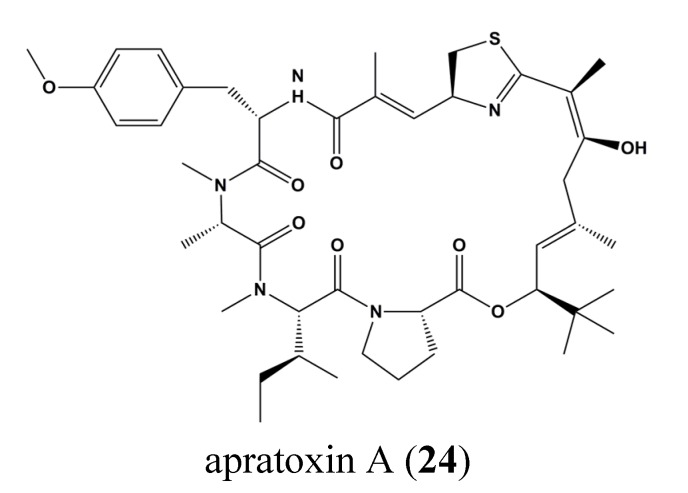
Chemical structure of apratoxin A (**24**).

Apratoxin A (**24**) (Figure 10) is a cytotoxic marine natural product that rapidly inhibits STAT-3 phosphorylation. Apratoxin A (**24**) inhibits interleukin 6 induced activation of JAK/STAT signaling and prevents N-glycosylation of receptor tyrosine kinases triggering proteasomal degradation. Moreover, the authors used proteomics to demonstrate down-regulation of proteins in the endoplasmic reticulum where N-glycoprotein are synthesized. By *in vitro* cell free systems, apratoxin A (**24**) was shown to prevent co-translational translocation of proteins of the secretory pathway [85].

**Figure 11:**
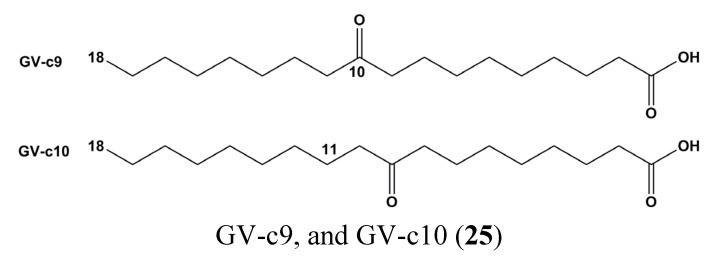
Chemical structures of two enone fatty acids, GV-c9, and GV-c10 (**25**).

Two enone fatty acids, GV-c9, and GV-c10 (**25**) (Figure 11) from *Gracilaria verrucosa*, a marine red alga with anti-oxidant and anti-cancer properties, inhibited NF-κB reporter activity by blocking NF-κB nuclear translocation as well as JAK/STAT (p-STAT1) signaling [86].

## 5. Conclusions and Perspectives

In the field of marine compound JAK/STAT inhibitors, we feel that much remains to be discovered. Even though the number of *bona fide* JAK/STAT inhibitors remains modest, we strongly believe that many inhibitors so far identified as NF-κB inhibitors of marine origins could also act as JAK/STAT inhibitors considering the fact that many compounds from terrestrial plants showed significant redundancy concerning the inhibition of both key inflammation pathways. Active research in this direction could lead to additional discoveries. Finally, both NF-κB and STAT transcription factors are regulated by epigenetic post-translational regulatory mechanisms. Considering the important number of epigenetically active molecules also from marine origins, we speculate here in regards to marine compounds that would confer epigenetic regulation whether at the level of DNA methylation, histone modifications or small regulatory RNA expression, and hypothesize that marine natural compounds could regulate the JAK/STAT regulatory pathways in such a way [15,87,88].

## Abbreviations

ALL: Acute lymphocytic leukemia
AML: Acute myelogenous leukemia
Bcr-Abl: Break point cluster–Abelson
CLL: Chronic lymphocytic leukemia
DNA: Deoxyribonucleic Acid
EGFR: Epidermal growth factor receptor
EMEA: European Medicines Agency
EsSOCS: *Eriocheir sinensis* suppressors of cytokine signaling
INF: interferon
IL: interleukin
JAK: Janus kinase
JfGPH: Japanese flounder glycoprotein 130 homologue
JNK: c-Jun N-terminal kinase
NCI: National Cancer Institute
NF-κB: nuclear factor-κB
p53: tumor suppressor protein 53
RbSTAT4: Single transducer and activator of transcription 4 family identified from rock bream
ROS: reactive oxygen species
SD: Sarcodiol
SmSOCS3: *Scophthalmus maximus* suppressors of cytokine signaling 3
SOCS2: suppressors of cytokine signaling 2
STAT: Single transducer and activator of transcription
WHO: World Health Organization
WSSV: White spot syndrome virus
